# Rift Valley Fever Outbreak in Livestock, Mozambique, 2014

**DOI:** 10.3201/eid2212.160310

**Published:** 2016-12

**Authors:** José M. Fafetine, Peter Coetzee, Benjamin Mubemba, Ofélia Nhambirre, Luis Neves, J.A.W. Coetzer, Estelle H. Venter

**Affiliations:** Eduardo Mondlane University, Maputo, Mozambique (J.M. Fafetine, O. Nhambirre);; University of Pretoria, Pretoria, South Africa (P. Coetzee, L. Neves, J.A.W. Coetzer, E.H. Venter);; Copperbelt University, Kitwe, Zambia (B. Mubemba)

**Keywords:** Rift Valley fever, Rift Valley fever virus, ruminants, abortion, PCR, ELISA, Mozambique, viruses, livestock, vector-borne infections

## Abstract

In early 2014, abortions and death of ruminants were reported on farms in Maputo and Gaza Provinces, Mozambique. Serologic analysis and quantitative and conventional reverse transcription PCR confirmed the presence of Rift Valley fever virus. The viruses belonged to lineage C, which is prevalent among Rift Valley fever viruses in southern Africa.

Rift Valley fever (RVF) virus (family *Bunyaviridae*, genus *Phlebovirus*) is a mosquito-borne virus that affects ruminants and humans. The virion contains 3 single-stranded RNA genome segments, large, medium, and small. In ruminants, RVF virus infection is characterized by high rates of abortion and of death, particularly in newborn animals. In humans, the infection is usually asymptomatic, but in severe cases, hemorrhage, meningoencephalitis, retinopathy, and death can occur (*1*).

Some of the most notable RVF epidemics reported in the past 2 decades occurred in eastern Africa and in southern Africa, where Mozambique is located. In 2006 and 2007, outbreaks of the disease occurred in eastern Africa, including Tanzania (*2*). In 2008 (*3*) and 2010 (*4*), epidemics of the disease were reported in South Africa. However, during the same period, no RVF outbreaks were reported in Mozambique. The few confirmed RVF outbreaks in the country occurred in 1969 in Gaza and Maputo Provinces, resulting in the deaths of 220 and 25 cattle in each province, respectively (*5*). In 1999, cases of abortion in a herd of water buffaloes (*Bubalus bubalis*) in Zambézia Province were attributed to RVF virus, but no virus was detected or isolated (*6*).

In 2010, serosurveys were conducted in the Zambézia and Maputo Provinces of Mozambique. Seroprevalences of 9.2% in sheep and 11.6% in goats was recorded in Zambézia Province (*7*). In Maputo Province, an overall seroprevalence of 36.9% was documented in cattle (*8*). The results indicated the possible circulation of RVF virus during interepidemic periods without the manifestation of typical clinical signs, as has been described elsewhere (*9*).

In this article, we report the detection of specific antibodies against RVF virus and the genetic analysis of RVF virus isolates from outbreaks in Mozambique. We also discuss the possible factors associated with the occurrence of this outbreak.

## The Study

In late March 2014, after a period of heavy and persistent rainfall in southern Mozambique, particularly in Maputo and Gaza Provinces, abortions and deaths in ruminant offspring were reported on some farms. The owner of a farm located in the Goba District, Maputo Province (26°3′59.73′′S, 32°0′23.36′′E), informed the veterinary authorities that 16 of 88 goats aborted their fetuses and 5 newborn kids died. The veterinary authorities also received reports from 2 farms in Xai-Xai (25° 03′24.1”S, 33°41′24.7”E) and Chibuto (24°42′01.2′′S, 33°32′24.8′′E) in Gaza Province, where 26 goats and 8 sheep aborted, respectively, and a total of 7 newborn animals died on both farms. According to the farmers, no animals had been purchased or brought into the herds for >3 years.

Serum samples were collected from farms in Goba (n = 88), Xai-Xai (n = 26), and Chibuto (n = 13). On the Goba farm, liver and spleen tissue samples were also collected from 1 aborted fetus and from 1 dead newborn goat. All the serum samples were tested for the presence of RVF virus IgM by using the IDvet Screen RVF IgM ELISA (IDvet innovative diagnostics kit; IDVet, Montpellier, France). In addition, the serum samples collected in Goba were further tested for RVF virus IgG by using the RVF recN IgG ELISA kit (Biological Diagnostic Supplies Limited, Edinburgh, Scotland, UK).

Viral genomic RNA was extracted from ELISA-positive serum samples and tissue samples by using Trizol (Invitrogen, Manchester, UK) according to the manufacturer’s instructions. A quantitative real-time reverse transcription PCR was performed as described (*10*). Positive samples were subsequently subjected to a conventional RT-PCR to amplify a 490-nt region of the medium segment as described (*11*). The amplicons were then purified by using the QIAquick Gel extraction kit (QIAGEN, Manchester, UK) and submitted to Inqaba Biotec (Pretoria, South Africa) for sequencing. The obtained sequences were compared with sequences in GenBank using BLAST (https://blast.ncbi.nlm.nih.gov/Blast.cgi). Sequence data (480 nt) were imported into MEGA6 (*12*), in which sequence alignment and evolutionary analyses were carried out. The phylogenetic trees were inferred by using the maximum-likelihood method based on the Kimura 2-parameter model (*13*). The tree with the highest log likelihood (−2055.6526) is shown in the Technical Appendix Figure). Reference RVF virus isolates used in this study are listed in the Technical Appendix Table. 

Serologic analysis indicated that 31 (24.4%) of 127 (all) sampled animals were positive for RVF virus IgM, and 49 (55.7%) of 88 animals from the Goba Province were positive for RVF virus IgG. Only 25 animals had RVF virus IgG but no RVF virus IgM (Table). The data suggest that the onset of the outbreak on the Goba farm was in early or mid-January 2014 and continued until mid-April because at this time only a few RVF IgM–positive animals (2/26 animals) contained IgM but no IgG against RVF virus, and few abortions were continuing. RVF virus RNA was detected in 16 serum samples and 6 tissue samples analyzed by quantitative real-time RT-PCR, confirming an RVF outbreak in Mozambique. Only PCR products obtained from fetal tissue met the minimum concentration requirement for sequencing. Phylogenetic analysis showed that the Goba-Mozambique isolate belonged to the lineage C group of RVF viruses (Technical Appendix Figure).

**Table:** ELISA and RT-PCR results for sheep and goat serum specimens tested during Rift Valley Fever outbreak, Mozambique, 2014*

Results	Goba District	Xai-Xai District	Chibuto District
Total	88	26	13
No. IgM positive	26	2	3
No. IgG positive	49	NT	NT
No. IgM positive only	2	NA	NA
No. IgG positive only	25	NA	NA
No. RT-PCR positive	12	2	2
No. RT-PCR and IgM positive	1	2	2
No. RT- PCR and IgG positive	0	NA	NA
No. RT-PCR, IgM and IgG positive	11	NA	NA

*RVF, Rift Valley fever; RT-PCR, real-time quantitative reverse transcription PCR; NA, not available; NT, not tested.

## Conclusions

RVF virus IgM and the molecular detection of RVF virus confirmed the cause of abortions and deaths in sheep and goats in Maputo (Goba) and Gaza (Xai-Xai and Chibuto) Provinces of Mozambique in the first quarter of 2014. Outbreaks of RVF in eastern Africa are usually associated with the circulation of a local virus lineages, triggered by abnormal rainfall that favors the multiplication of the mosquito vectors or by the introduction of virus through animal movement. Sequence comparison and phylogenetic analyses indicated that the Maputo RVF viruses belonged to lineage C, which suggested that the outbreaks had close links to the 2007 and 2010 RVF outbreaks in Sudan. 

The farms where the outbreaks occurred are noncommercial, small- to middle-scale farming systems with basic management and with no reports of animal importation. Occasionally, animals are bought from other farms, and the animals known to be imported in the southern part of the country come from the neighboring countries (i.e., South Africa and Swaziland). Animal movement is also reported to occur frequently on the border with adjoining countries. Because no new animals were introduced onto the above mentioned farms and no animal importation was indicated either by the farmers or by the veterinary authorities, we hypothesize that the virus might have been introduced in the past. Then, after a 6-fold increase in rainfall in Maputo (31 mm in November 2013 and 208 mm in December 2013; Umbeluzi Weather Station, pers. comm.), the conditions for an increase in the vector population, and therefore in virus circulation, favored the occurrence of the outbreaks. The high level of seroprevalence reported previously in districts close to the study site (*8*) may suggest a continuous low level of virus transmission in the region that is exacerbated by above-average rainfall, resulting in RVF virus infection in naive animals. 

With this confirmed genetic evidence of RVF virus in Mozambique, all countries on the eastern coastline of Africa, the Indian Ocean islands of Madagascar and Mayotte, and Yemen and Saudi Arabia have all reported the presence of viruses belonging to lineage C (*11*). The broad geographic distribution pattern of lineage C viruses (in southern and northern Africa) and the related life-cycle dynamics require further investigation to identify the main drivers associated with the circulation and spread of this lineage of viruses.

Technical Appendix Reference Rift Valley fever (RVF) virus isolates used in this study and phylogenetic tree generated from sequence data obtained from RVF viruses from Goba District, Mozambique, and RVF reference sequences available in GenBank.
