# Supplementary material for: Rift Valley Fever Outbreak in Livestock, Mozambique, 2014
Source: Emerg Infect Dis. 2016 Dec;22(12):2165–7. doi: 10.3201/eid2212.160310 (PMC5189134; doi:10.3201/eid2212.160310)
Supplement: Technical Appendix — Reference Rift Valley fever (RVF) virus isolates used in this study and phylogenetic tree generated from sequence data obtained from RVF viruses from Goba District, Mozambique, and RVF reference sequences available in GenBank. [file 16-0310-Techapp-s1.pdf]

Article DOI: http://dx.doi.org.10.3201/eid2212.160310

Rift Valley Fever Outbreak in Livestock, Mozambique, 2014

Technical Appendix

Technical Appendix Table. Partial (490 nt) M-segment sequences of RVF virus isolates accessed from NCBI included in this study

No	Isolate	Country	Year of isolation	Reference	GenBank accession no.
1	SPU52/99/1	South Africa	1999	Grobbelaar et al. 2011	HM587046
2	SPU52/99/5	South Africa	1999	Grobbelaar et al. 2011	HM587047
3	SPU52/99/2	South Africa	1999	Grobbelaar et al. 2011	HM587048
4	SPU52/99/3	South Africa	1999	Grobbelaar et al. 2011	HM587049
5	SPU86/09	South Africa	2009	Grobbelaar et al. 2011	HM587065
6	SA152/08	South Africa	2008	Grobbelaar et al. 2011	HM587066
7	M37/08	South Africa	2008	Grobbelaar et al. 2011	HM587067
8	SA52/08	South Africa	2008	Grobbelaar et al. 2011	HM587069
9	An278	Saudi Arabia	2000	Grobbelaar et al. 2011	HM587050
10	SA01–1322	Saudi Arabia	2001	Miller et al. 2001	AF393745
11	H2MAU03	Mauritania	2003	Faye et al. 2003	EF160115
12	H1MAU03	Mauritania	2003	Faye et al. 2003	EF160116
13	SPU12/99/21	Somalia	1998	Grobbelaar et al. 2011	HM587051
14	SPU384/97/1	Kenya	1997	Grobbelaar et al. 2011	HM587052
15	SPU2/98/1	Kenya	1998	Grobbelaar et al. 2011	HM587053
16	SPU2/98/9	Kenya	1998	Grobbelaar et al. 2011	HM587055
17	SPU22/07/118	Kenya	2007	Grobbelaar et al. 2011	HM587062
18	SPU22/07/125	Kenya	2007	Grobbelaar et al. 2011	HM587063
19	SPU22/07/129	Kenya	2007	Grobbelaar et al. 2011	HM587064
20	SPU22/07/4	Kenya	2007	Grobbelaar et al. 2011	HM587068
21	B309	Kenya	1977	Grobbelaar et al. 2011	HM587070
22	214445	Kenya	1983	Grobbelaar et al. 2011	HM587074
23	B1143	Kenya	1977	Grobbelaar et al. 2011	HM587075
24	VRL2413/98	Zimbabwe	1998	Grobbelaar et al. 2011	HM587054
25	VRL1187/79	Zimbabwe	1979	Grobbelaar et al. 2011	HM587056
26	VRL2354/78	Zimbabwe	1978	Grobbelaar et al. 2011	HM587058
27	VRL1548/78	Zimbabwe	1978	Grobbelaar et al. 2011	HM587059
28	VRL825/79	Zimbabwe	1979	Grobbelaar et al. 2011	HM587071
29	VRL2051/76	Zimbabwe	1976	Grobbelaar et al. 2011	HM587072
30	VRL1217/78	Zimbabwe	1978	Grobbelaar et al. 2011	HM587073
31	VRL1290/78	Zimbabwe	1978	Grobbelaar et al. 2011	HM587077
32	MgAn1002	Madagascar	1991	Grobbelaar et al. 2011	HM587057
33	MgAn991	Madagascar	1991	Grobbelaar et al. 2011	HM587060
34	MgAn990	Madagascar	1991	Grobbelaar et al. 2011	HM587061
35	M48	Madagascar	2008		HQ009512
36	SPU204/85	Angola	1985	Grobbelaar et al. 2011	HM587076
37	FI-2	Madagascar	2008	Ratovonjato et al. 2011	GU135856
38	AL-53	Madagascar	2008	Ratovonjato et al. 2011	GU135862
39	MgH824	Madagascar	1979	Grobbelaar et al. 2011	HM587040
40	93-Abeer	Egypt	1993	Grobbelaar et al. 2011	HM587043
41	SPU44/85	Zambia	1985	Grobbelaar et al. 2011	HM587079
42	74HB59	CAR	1974	Grobbelaar et al. 2011	HM587082
43	SA373	South Africa	2010	Grobbelaar et al. 2011	HM587097
44	SPU77/04	Namibia	2004	Grobbelaar et al. 2011	HM587100
45	Ar20364	South Africa	1981	Grobbelaar et al. 2011	HM587101
46	SA55	South Africa	1955	Grobbelaar et al. 2011	HM587120
47	SA51	South Africa	1951	Grobbelaar et al. 2011	HM587125
48	M34_ARB	Senegal	2002	Soumare et al. 2012	JN995327
49	M38_ARB	Senegal	2003	Soumare et al. 2012	JN995343
50	Namibia_2010	Namibia	2010	Monaco et al. 2013	KC935380
51	211977	Mauritania	2010	Faye et al. 2013	KF717590

No	Isolate	Country	Year of isolation	Reference	GenBank accession no.
52	ZH501	Egypt	1977	Bird et al. 2007	DQ380200
53	ZH548	Egypt	1977	Bird et al. 2007	DQ380206
54	ZC3349	Egypt	1978	Bird et al. 2007	DQ380207
55	ZM657	Egypt	1978	Bird et al. 2007	DQ380204
56	ZS6365	Egypt	1979	Bird et al. 2007	DQ380205
57	ZH1776	Egypt	1978	Bird et al. 2007	DQ380203
58	T-46	Egypt	1977	Bird et al. 2007	DQ380199
59	73HB1230	CAR	1973	Bird et al. 2007	DQ380221
60	Zinga	CAR	1969	Bird et al. 2007	DQ380217
61	CAR-R1622	CAR	1985	Bird et al. 2007	DQ380219
62	73HB1449	CAR	1973	Bird et al. 2007	DQ380211
63	74HB59	CAR	1974	Bird et al. 2007	DQ380212
64	Hv-B375	CAR	1985	Bird et al. 2007	DQ380218
65	2250/74	Zimbabwe	1974	Bird et al. 2007	DQ380209
66	1853/78	Zimbabwe	1978	Bird et al. 2007	DQ380220
67	1260/78	Zimbabwe	1978	Bird et al. 2007	DQ380214
68	763/70	Zimbabwe	1970	Bird et al. 2007	DQ380188
69	2373/74	Zimbabwe	1974	Bird et al. 2007	DQ380194
70	2269/74	Zimbabwe	1974	Bird et al. 2007	DQ380222
71	MgH824	Madagascar	1979	Bird et al. 2007	DQ380210
72	200803162	Madagascar	2008	Carrol et al. 2011	JF311377
73	200803167	Madagascar	1991	Carrol et al. 2011	JF311382
74	200803166	Madagascar	1991	Carrol et al. 2011	JF311381
75	200803163	Madagascar	2008	Carrol et al. 2011	JF311378
76	200803169	Madagascar	2008	Carrol et al. 2011	JF311384
77	200803170	Madagascar	2008	Carrol et al. 2011	JF311385
78	21445	Kenya	1983	Bird et al. 2007	DQ380198
79	9800523	Kenya	1998	Bird et al. 2007	DQ380196
80	IB8	Kenya	1965	Bird et al. 2007	DQ380190
81	KEN006/07	Kenya	2007	Nderitu et al. 2011	HM586966
82	KEN032/07	Kenya	2007	Nderitu et al. 2011	HM586967
83	2007000260	Kenya	2006	Bird et al. 2012	JF326193
84	2007002820	Kenya	2007	Bird et al. 2008	JF309200
85	Saudi10911	Saudi Arabia	2000	Bird et al. 2007	DQ380197
86	ANK3837	Guinea	1981	Bird et al. 2007	DQ380215
87	ANK6087	Guinea	1984	Bird et al. 2007	DQ380216
88	OS-1	Mauritania	1987	Bird et al. 2007	DQ380186
89	OS-3	Mauritania	1987	Bird et al. 2007	DQ380184
90	OS-9	Mauritania	1987	Bird et al. 2007	DQ380183
91	OS-8	Mauritania	1987	Bird et al. 2007	DQ380185
92	ArD38388	Burkina Faso	1983	Bird et al. 2007	DQ380187
93	Entebbe	Uganda	1944	Bird et al. 2007	DQ380191
94	SA75	South Africa	1975	Bird et al. 2007	DQ380189
95	SA51	South Africa	1951	Bird et al. 2007	DQ380195
96	Kakamas	South Africa	2009	Potgieter et al. 2011	JQ068143
97	2008/00099	Mayotte	2008	Cetre-Sossah et al. 2012	HE687303
98	2008/00101	Mayotte	2008	Cetre-Sossah et al. 2012	HE687306
99	2007000323	Tanzania	2007	Bird et al. 2011	JF326194
100	2007000324	Tanzania	2007	Bird et al. 2011	JF326195
101	TAN001/O7	Tanzania	2007	Nderitu et al. 2011	HE586970
102	TAN002/07	Tanzania	2007	Nderitu et al. 2011	HE586971
103	7–2010	Sudan	2010	Aradaib et al. 2013	JQ820487
104	85–2010	Sudan	2010	Aradaib et al. 2013	JQ820488
105	86–2010	Sudan	2010	Aradaib et al. 2013	JQ820489
106	2V-2007	Sudan	2007	Aradaib et al. 2013	JQ820490
107	28–2010	Sudan	2010	Aradaib et al. 2013	JQ820491

References

1. Aradaib IE, Erickson BR, Elageb RM, Khristova ML, Carroll SA, Elkhidir IM, et al. Rift Valley fever, Sudan, 2007 and 2010. [Epub 2013 Feb]. Emerg Infect Dis. 2013;19:246–53. http://dx.doi.org/10.3201/eid1902.120834

2. Bird BH, Khristova ML, Rollin PE, Ksiazek TG, Nichol ST. Complete genome analysis of 33 ecologically and biologically diverse Rift Valley fever virus strains reveals widespread virus movement and low genetic diversity due to recent common ancestry. [Epub 2006 Dec 27]. J Virol. 2007;81:2805–16. PubMed http://dx.doi.org/10.1128/JVI.02095-06

3. Bird BH, Githinji JWK, Macharia JM, Kasiiti JL, Muriithi RM, Gacheru SG, et al. Multiple virus lineages sharing recent common ancestry were associated with a large Rift Valley fever outbreak among livestock in Kenya during 2006–2007. [Epub 2008 Sep 10]. J Virol. 2008;82:11152–66. PubMed http://dx.doi.org/10.1128/JVI.01519-08

4. Bird BH, Khristova ML, Nichol ST. Direct submission. Submitted 2011 Feb 11. Viral Special Pathogens Branch, National Center for Emerging and Zoonotic Infectious Diseases, Centers for Disease Control and Prevention, Atlanta, Georgia, USA. http://www.ncbi.nih.gov/nuccore/JF326194

5. Bird BH, Khristova ML, Nichol ST. Direct submission. Submitted 2011 Feb 11. Viral Special Pathogens Branch, National Center for Emerging and Zoonotic Infectious Diseases, Centers for Disease Control and Prevention, Atlanta, Georgia, USA. http://www.ncbi.nih.gov/nuccore/JF3261945

6. Carroll SA, Reynes JM, Khristova ML, Andriamandimby SF, Rollin PE, Nichol ST. Genetic evidence for Rift Valley fever outbreaks in Madagascar resulting from virus introductions from the East African mainland rather than enzootic maintenance. [Epub 2011 Apr 20]. J Virol. 2011;85:6162–7. PubMed

7. Cêtre-Sossah C, Zeller H, Grandadam M, Caro V, Pettinelli F, Bouloy M, et al. Genome analysis of Rift Valley fever virus, Mayotte. Emerg Infect Dis. 2012;18:969–71. PubMed

8. Faye O, Diallo M, Diop D, Elmamy BO, Ba H, Niang M, et al. Outbreak of Rift Valley Fever in Mauritania, 2003: First emergence of the East-Central Africa virus lineage in West Africa. http://www.ncbi.nih.gov/nuccore/EF160115

9. Faye O, Ba H, Ba Y, Freire C, Faye O, Ndiaye O, et al. Direct submission. Submitted 2013 Oct 9. Arbovirus Unit, Institut Pasteur-Dakar, Senegal. http://www.ncbi.nih.gov/nuccore/KF717590

10. Grobbelaar AA, Weyer J, Leman PA, Kemp A, Paweska JT, Swanepoel R. Molecular epidemiology of Rift Valley fever virus. Emerg Infect Dis. 2011;17:2270–6 PubMed http://dx.doi.org/10.3201/eid1712.111035.

11. Miller BR, Godsey MS, Crabtree MB, Al-Mazrao Y, Al-Jeffri MH, Abdoon AM, et al. Direct submission. Submitted 2001 Jun 11. Division of Vector-Borne Infectious Diseases, National Center for Emerging and Zoonotic Infectious Diseases, Centers for Disease Control and Prevention, Atlanta, Georgia, USA. http://www.ncbi.nih.gov/nuccore/AF393745

12. Monaco F, Pinoni C, Cosseddu GM, Khaiseb S, Calistri P, Molini U, et al. Rift Valley fever in Namibia, 2010. Emerg Infect Dis. 2013;19:2025–7. PubMed http://dx.doi.org/10.3201/eid1912.130593

13. Nderitu L, Lee JS, Omolo J, Omulo S, O'Guinn ML, Hightower A, et al. Sequential Rift Valley fever outbreaks in eastern Africa caused by multiple lineages of the virus. [Epub 2010 Dec 14]. J Infect Dis. 2011;203:655–65. PubMed http://dx.doi.org/10.1093/infdis/jiq004

14. Potgieter CA, Smith SJ, Kortekaas J. [Virology, Central Veterinary Institute of Wageningen, University and Research Centre, Lelystad, the Netherlands.]. Direct submission. 2011. http://www.ncbi.nih.gov/nuccore/JQ068143

15. Ratovonjato J, Olive MM, Tantely LM, Andrianaivolambo L, Tata E, Razainirina J, et al. Detection, isolation, and genetic characterization of Rift Valley fever virus from *Anopheles* (*Anopheles*) *coustani*, *Anopheles* (*Anopheles*) *squamosus*, and *Culex* (*Culex*) *antennatus* of the Haute Matsiatra region, Madagascar. [Epub 2010 Oct 28]. Vector Borne Zoonotic Dis. 2011;11:753–9. PubMed http://dx.doi.org/10.1089/vbz.2010.0031

16. Soumaré POL, Freire CCM, Faye O, Diallo M, de Oliveira JV, Zanotto PM, et al. Phylogeography of Rift Valley Fever virus in Africa reveals multiple introductions in Senegal and Mauritania. PLoS One. 2012;7:e35216. PubMed http://dx.doi.org/10.1371/journal.pone.0035216

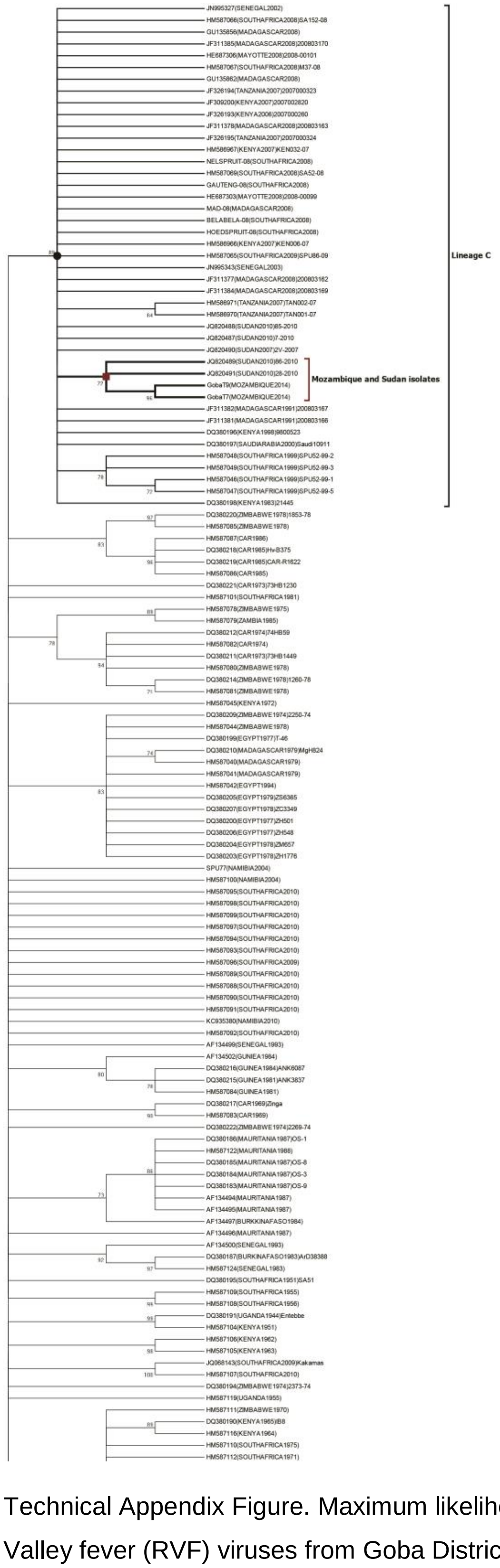

Technical Appendix Figure. Maximum likelihood tree generated from sequence data obtained from Rift Valley fever (RVF) viruses from Goba District, Mozambique, and RVF reference sequences available on Genbank (n = 129;480 nt region; medium segment). The evolutionary history was inferred by using the

maximum-likelihood method in MEGA6 (*12*), based on the Kimura 2-parameter model. The tree that had the highest log likelihood (–2055.6526) is shown in the figure. A discrete γ distribution was used to model the evolutionary rate differences among sites (5 categories [(+G, parameter = 0.3911]). Confidence values for the tree topologies were evaluated with a bootstrap analysis of 1,000 replicate datasets (consensus tree; cutoff <70% shown). Lineages were identified as described by Grobbelaar et al. (*10*).
